# Characterization of manganese superoxide dismutase from a marine cyanobacterium *Leptolyngbya valderiana *BDU20041

**DOI:** 10.1186/1746-1448-6-6

**Published:** 2010-06-03

**Authors:** Balakrishnan Priya, Reddi K Sivaprasanth, Vincent Dhivya Jensi, Lakshmanan Uma, Gopalakrishnan Subramanian, Dharmar Prabaharan

**Affiliations:** 1 National Facility for Marine Cyanobacteria (Sponsored by Dept. of Biotechnology, Govt. of India), Department of Marine Biotechnology, School of Marine Sciences, Bharathidasan University, Tiruchirappalli, Tamil Nadu, India; 2 Graduate Institute of Biotechnology, National Chung Hsing University, 250, Kuo-Kuang Road, Taichung, Taiwan

## Abstract

**Background:**

Cyanobacteria are recognized as the primordial organisms to grace the earth with molecular oxygen ~3.5 billion years ago as a result of their oxygenic photosynthesis. This laid a selection pressure for the evolution of antioxidative defense mechanisms to alleviate the toxic effect of active oxygen species (AOS) in cyanobacteria. Superoxide dismutases (SODs) are metalloenzymes that are the first arsenal in defense mechanism against oxidative stress followed by an array of antioxidative system. Unlike other living organisms, cyanobacteria possess multiple isoforms of SOD. Hence, an attempt was made to demonstrate the oxidative stress tolerance ability of marine cyanobacterium, *Leptolyngbya valderiana *BDU 20041 and to PCR amplify and sequence the SOD gene, the central enzyme for alleviating stress.

**Result:**

*L. valderiana *BDU 20041, a filamentous, non-heterocystous marine cyanobacterium showed tolerance to the tested dye (C.I. Acid Black 1) which is evident by increased in biomass (i.e.) chlorophyll *a*. The other noticeable change was the total ROS production by culture dosed with dye compared to the control cultures. This prolonged incubation showed sustenance, implying that cyanobacteria maintain their antioxidant levels. The third significant feature was a two-fold increase in SOD activity of dye treated *L. valderiana *BDU20041 suggesting the role of SOD in alleviating oxidative stress *via *Asada-Halliwell pathway. Hence, the organism was PCR amplified for SOD gene resulting in an amplicon of 550 *bp*. The sequence analysis illustrated the presence of first three residues involved in motif; active site residues at H4, 58 and D141 along with highly conserved Mn specific residues. The isolated gene shared 63.8% homology with MnSOD of bacteria confirmed it as Mn isoform. This is the *hitherto *report on SOD gene from marine cyanobacterium, *L. valderiana *BDU20041 of Indian subcontinent.

**Conclusion:**

Generation of Reactive Oxygen Species (ROS) coupled with induction of SOD by marine cyanobacterium, *L. valderiana *BDU20041 was responsible for alleviating stress caused by an azo dye, C. I. Acid Black 1. The partial SOD gene has been sequenced and based on the active site, motif and metal specific residues; it has been identified as Mn metalloform.

## Introduction

Cyanobacteria, the oxygen-evolving photosynthetic prokaryote originated ~3.5 billion years ago, occupy a credential position between pro- and eukaryotes [1]. The resultant tandem operation of two photosystems is now known as oxygenic or plant-type photosynthesis [1]. This marked the turning point in the evolution of earth, opening up the era for aerobes. For the survival of cyanobacteria with oxygenic photosynthesis, the selection pressure led to the evolution of SODs [2-7]. Cyanobacteria are well documented for its ability to maintain the antioxidant levels by releasing H_2_O_2 _into the environment [2-5]. The prime armory for the release of H_2_O_2 _is superoxide dismutases. The first implication on the protective role of cyanobacterial SOD in photo-oxidative damage was shown in *Anacystis nidulans *[6].

SODs are generally classified according to their metal species which acts as redox-active center to catalyze the dismutation reaction O_2_^- ^+ 2H^+ ^→ O_2 _+ H_2_O_2_. Cu/ZnSOD type consists of Cu(II) and Zn(II) at the active site, MnSOD has Mn(III), the third FeSOD possess Fe(III), and a fourth NiSOD contains Ni(II/III). Generally SODs are localization specific. MnSOD is found in the cytosol and thylakoid membrane, whereas Fe and NiSOD in the cytosol and Cu/Zn is periplasmic in location but cyanobacteria have multiple forms of each type encoded by more than one gene [7]. The existence of multiple SODs may result from the fact that the cells of cyanobacteria are divided into compartments by internal membranes [8]. Since O_2_^- ^ions are negatively charged and cannot cross the phospholipids bilayer readily, they are efficiently trapped within the compartment where they are generated. This may have been selected for the evolution of multiple SODs in compartmentalized cells [9].

Different levels of expressions in terms of SOD isoenzymes resulted in response to oxidative stress caused by various abiotic factors such as, pesticides [10], lignin and its model dye [11] and nutrient limitation [12] particularly of Fe/MnSOD. In addition, our *hitherto *reports on marine cyanobacterium, *Leptolyngbya valderiana *BDU 20041 on exposure to synthetic dye has shown that the organism decolourized Acid black1, C.I. 20470 (100 mG L^-1^) to 85.6% in 12 days [13,14]. The active moiety involved in decolourization was adjudged to be AOS, mainly H_2_O_2 _(40 μM of H_2_O_2 _mG^-1 ^Chl h^-1^) which is excreted by the organism into the *milieu *[14]. The SOD activity was found to be induced by two-fold in the presence of dye suggesting a significant role in the production of H_2_O_2 _[14].

In the present study, we proposed to analyze the role of SOD in *L. valderiana *BDU 20041 during oxidative stress at the molecular level in order to more precisely identify its isoform. Hence, an attempt was made to PCR amplify, sequence and identify the metalloform of the SOD from the marine cyanobacterium, *L. valderiana *BDU20041 of Indian subcontinent.

## Results and Discussions

ROS are repeatedly reported to be the inevitable by-products of biological redox reaction and normal metabolism in humans, animals, plants and algae [15]. Cyanobacteria are oxygenic photosynthetic organisms that are prone to the oxidative stress due to the facts that they contain an array of photosynthetic pigments and that they both produce oxygen during photosynthesis and consume oxygen during respiration. It has been estimated that 1% of O_2 _consumed by cells is diverted to produce ROS in various subcellular loci [16].

The increased production of ROS and the resultant oxidative stress are considered to be the initial event and act as an alert signal for the organisms under several environmental stresses, such as light, temperature and UV. Inevitably, ROS also react with biologically important molecules such as lipids, proteins and DNA, inducing oxidative damage in membranes and the photosynthetic apparatus, which probably results in the death of cells [15]. Here we focus on synthetic dye induced oxidative stress and oxidative damage in cyanobacteria.

**Figure 1:**
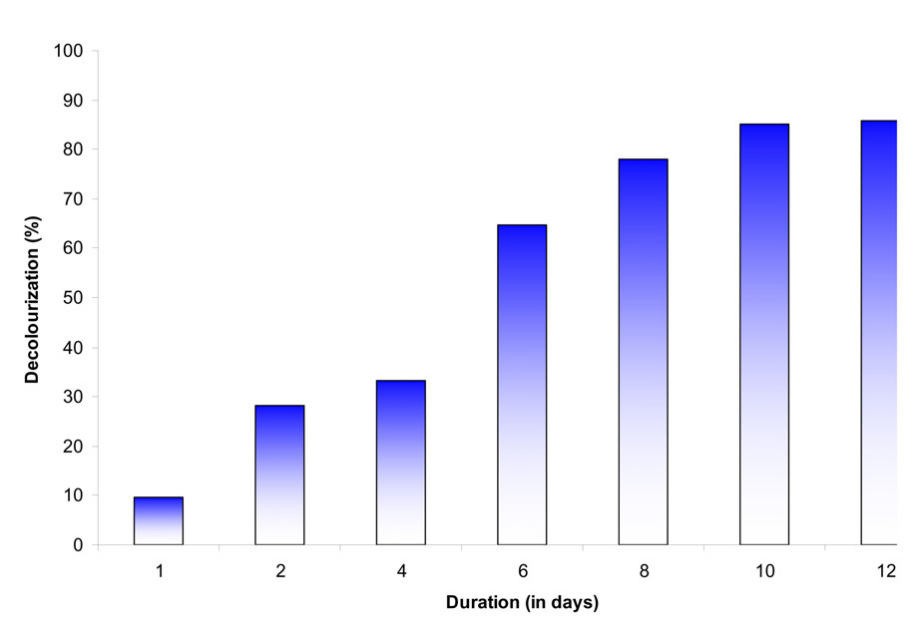
**Rate of decolourization of C.I. Acid Black 1 (100 mG L^-1^) by *L. valderiana *BDU20041 in ASN III nitrogen free medium**.

In the present study, the dye dosed stress response of *L. valderiana *BDU20041 was tested in nitrogen free ASN III medium for a stipulated period of 12 days. The response to stress was determined by the organism's decolourizing efficiency which varied among the duration tested (Figure 1). This shows that decolourization was only due to the metabolic activity of the organisms and not an abiotic factor. The results compliance with the decolourization studies by cyanobacteria [17,18].

Earlier reports deciphers that oxidative stress induces formation of ROS by multiple pathways [15,19]. Firstly, the photosynthetic pigments such as chlorophylls and phycobilins in cyanobacteria that act as photosensitizers (PS) under stress [15]. It is noteworthy that the energy of excited chlorophylls or phycobilins is utilized efficiently for photosynthesis under normal growth conditions. However, the inhibition of photosynthesis or the electron transport chain under stress may elevate the photosensitization process as well as the formation of ROS in this way.

Secondly, the essential role of the photosynthetic electron transport chain in the life of cyanobacteria promotes the possibility of subjection to oxidative stress. The probable electron transfer from the electron transport chain, especially in photosystem I (PSI), to molecular oxygen, the way to quench extensive excitation energy, is an alternative source of ROS. Photoreduction of molecular oxygen by the primary electron acceptor in the PSI complex is thought to be the main source of superoxide in illuminated cells [19].

Hence in the present study, experiments were carried out to evaluate the above phenomena as a response of *L. valderiana *BDU20041 to oxidative stress caused by C.I. Acid Black 1.

**Figure 2:**
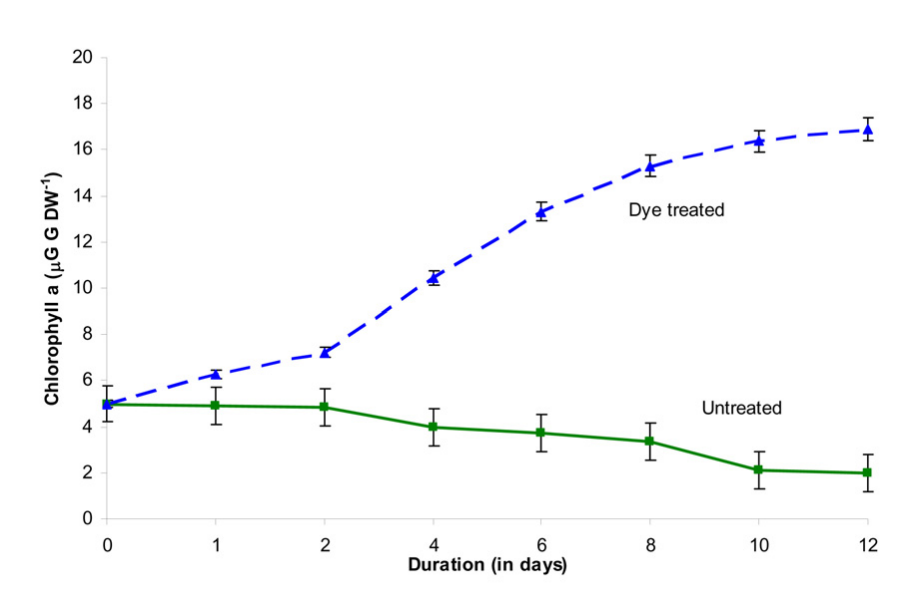
**Effect of C.I. Acid Black 1 on chlorophyll *a *content of *L. valderiana *BDU20041 in ASN III nitrogen free medium**.

**Figure 3:**
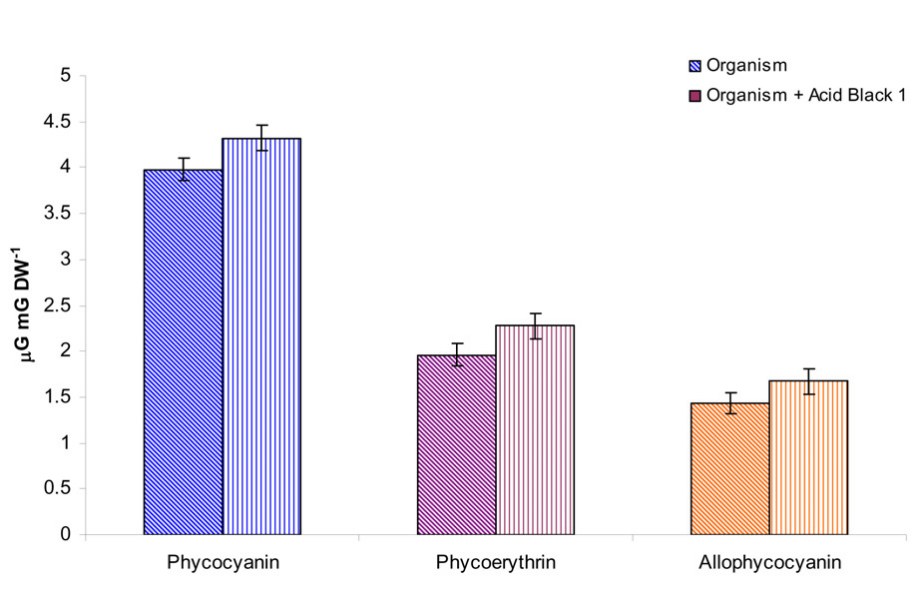
**Effect of C.I. Acid Black 1 on phycobilins content of *L. valderiana *BDU20041 in ASN III nitrogen free medium at the end of 12 days**.

First, dye treated *L. valderiana *BDU20041 showed increase in chlorophyll *a *proportional to the rate of decolourization (Figure 2) which suggests that dye hinders the availability of light for the photosynthetic machinery. In addition, there was a slight variation in the phycobilins of dye untreated and treated *L. valderiana *BDU20041 at the end of 12 days (Figure 3). These changes in chlorophyll and phycobilins during decolourization corroborate with the first phenomena mentioned above on the organism's potential to strive against oxidative stress caused by C.I. Acid Black 1.

**Figure 4:**
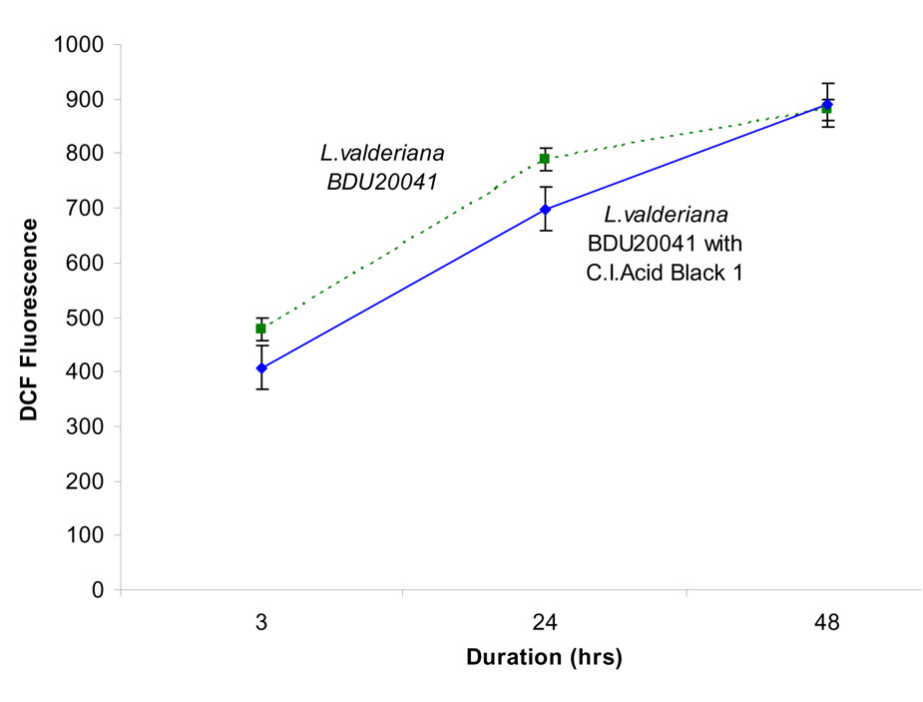
**Time course measurement of DCF oxidation (*invivo *ROS) by *L. valderiana *BDU20041 treated and untreated with C.I. Acid Black 1 (100 mG L^-1^)**.

The second notable feature is the total ROS produced in stress response which is represented as 2', 7'- Dichorofluorescein (DCF) with relative fluorescence intensity at 520 *nm*. Generally, ROS levels increase in response to abiotic stress [20]. Decreased levels of ROS initially in the 3^rd ^h with *L. valderiana *BDU20041 dosed with dye may be due to its involvement in degradation whereas, within 24 h, the organism increases the rate of ROS production and showed sustenance (Figure 4).

The initial decrease in total ROS in dye dosed culture could be attributed to two reasons (i) role of ROS in dye decolourization [21-23] (ii) the free radical generated passes through the Asada-Halliwell pathway initiated by SOD that converts the highly reactive oxy-radicals through an array of reaction to less toxic forms [19,24]. Further, the sustenance of ROS clearly depicts the stress response in *L. valderiana *BDU20041 as cyanobacteria maintain their antioxidants level by release of reactive oxygen species into the *milieu *[5,24]. Studies with eukaryotic and bacterial systems showed that, low levels of AOS are indispensable to act in cellular signaling and in the control of gene expression [25]. Because of the dual functions of ROS, a tight control of their concentrations may be anticipated, which requires a delicate balance of systems involved in their generation and destruction [26].

**Figure 5:**
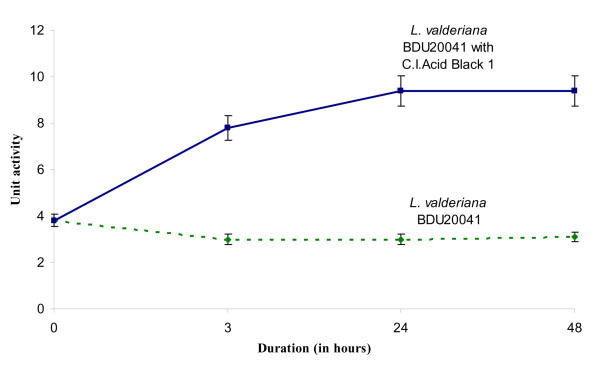
**Time course measurement on effect of C.I. Acid Black 1 on superoxide dismutase activity of *L. valderiana *BDU20041 in ASN III nitrogen free medium**.

Hence, the third significant trait studied was the response of SOD enzyme to oxidative stress (in this case, C.I. Acid Black 1). SOD activity was found to increase in the 3^rd ^h and on further exposure to dye, the activity increased two-fold and got sustained thereafter (Figure 5). This obviously proves that SOD plays a significant role in alleviating oxidative stress in *L. valderiana *BDU20041.

As the initial oxy-radical product to be formed under any oxidative stress is the superoxide radical (O_2_^-.^) which upon further reaction within the cell can generate more ROS such as hydroxyl radicals and singlet oxygen. Superoxide dismutases are metalloenzymes that dismutase these superoxide radical to hydrogen peroxides. Their activity increases on exposure to oxidative stress [16]. Hence, they can be depicted as the central dogma of antioxidative system. Hence, in our study attempts was made to isolate and characterize the SOD gene involved in abating oxidative stress caused by C.I. Acid Black 1.

**Figure 6:**
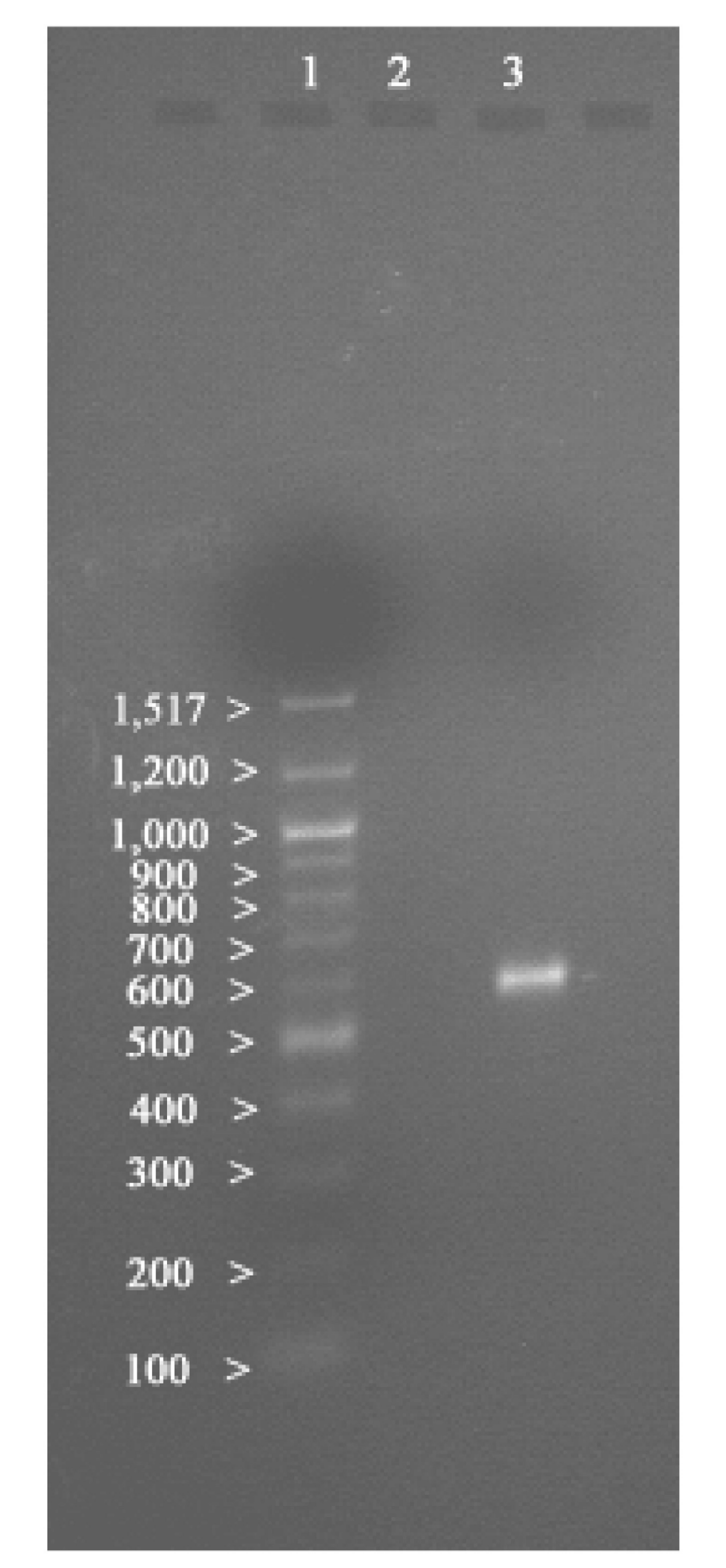
**Agarose gel electrophorogram (1.5%) showing an amplified MnSOD product of 550 bp from *Leptolyngbya valderiana *BDU20041**. Lane1- 1 Kb marker, Lane 2- Negative control, Lane 3- Amplified PCR product.

**Figure 7:**
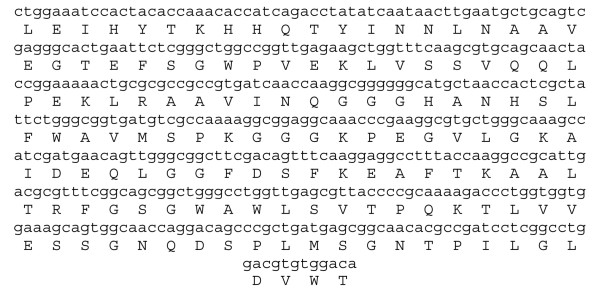
**Nucleotide Sequence for MnSOD of *Leptolyngbya valderiana *BDU20041**. Shown below nucleotide (AY974247) is the translated protein (AAX84682) sequence used for structure determination.

The SOD gene from the DNA isolated of marine cyanobacterium, *L. valderiana *BDU20041 was amplified using the below mentioned primers. Electrophoresis of the amplified products of gradient PCR (61°C) showed a band of 550 *bp *and none in negative control (Figure 6). The nucleotide sequence of the partial SOD gene of 432 *bp *was submitted to GenBank database (AY974247) and their deduced aminoacid was 144 residues (AAX84682) (Figure 7). The computational analysis on the sequenced partial SOD gene comprised of an N-terminal and a C-terminal region from 1 to 63 and 70 to 143 residues respectively with a theoretical molecular weight of 15.5 *KDa*.

When the obtained sequence was aligned within cyanobacterial SOD, MnSOD of *Thermosynechococcus elongates *BP1 (BAC07589) and *Leptolyngbya boryana *(P50058) had maximum homology of 47%. The least homology of 43% was shared with *Leptolyngbya boryana *(P50056). The homology difference could be attributable to partial SOD gene, in particular C-terminal region where most of the aminoacids are highly conserved residues along with motif region (DVWEHAYY).

**Figure 8:**
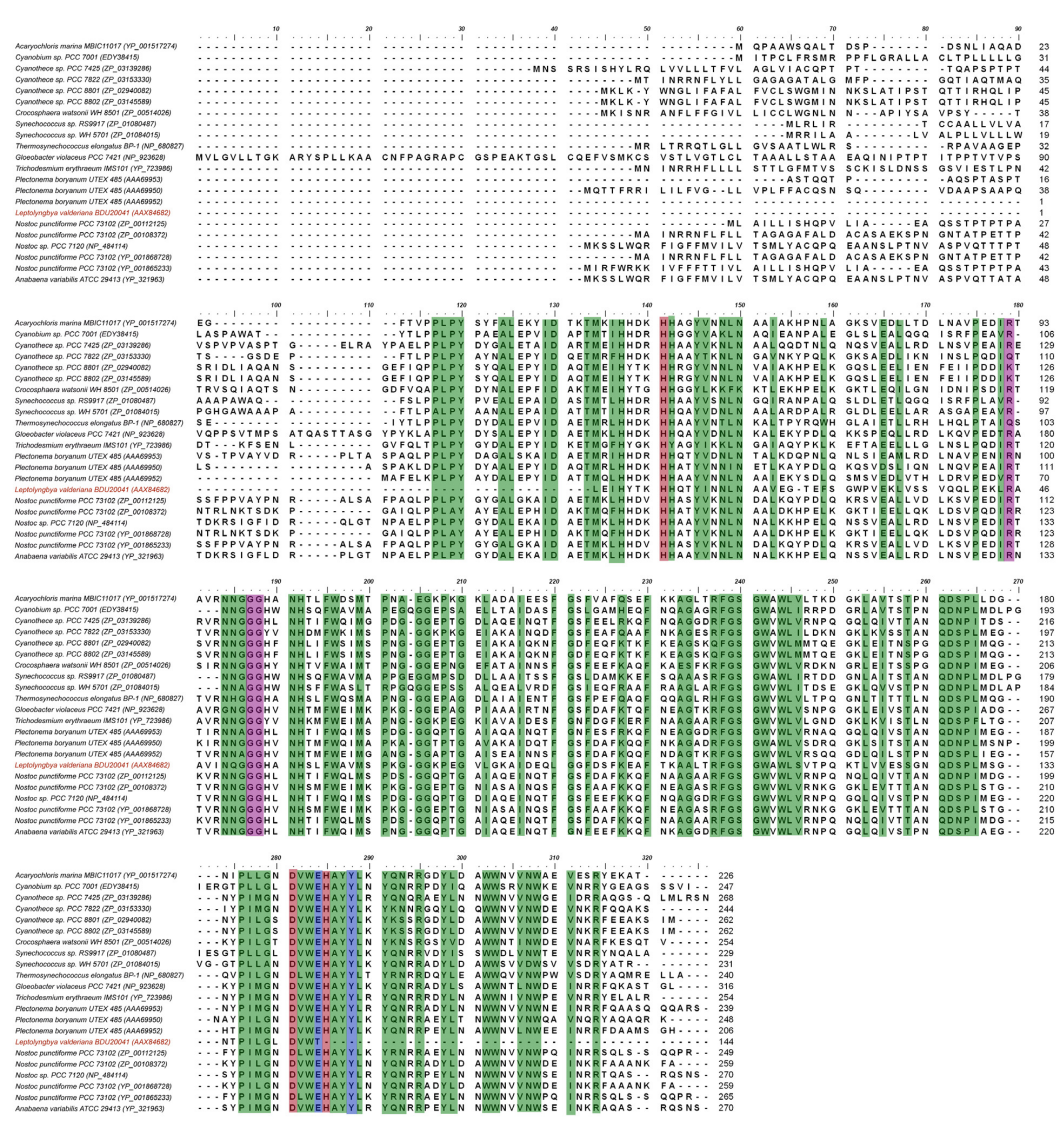
**Sequence alignment of cyanobacterial Manganese superoxide dismutase**. Alignment showing 95% conserved residues using Clustal W of BioEdit Package (v.7.0.5) [33] Residues involved in active site are highlighted in red, metal specific residues in pink and highly conserved residues for MnSOD in green.

Further, the isolated sequence on analysis (Figure 8) showed the presence of first 3 residues of SOD motif region DVWEHAYY (D_281_-W_223_) along with conserved residues-glycine (G_53_), histidine (H_55_), phenylalanine (F_61_), serine (S_105_), tryptophan (W_108_), leucine (L_109_), arginine (N_125_) and glutamine (Q_126_). These highly conserved residues were found to be precise for cyanobacterial MnSOD as described by Priya et al [7].

**Figure 9:**
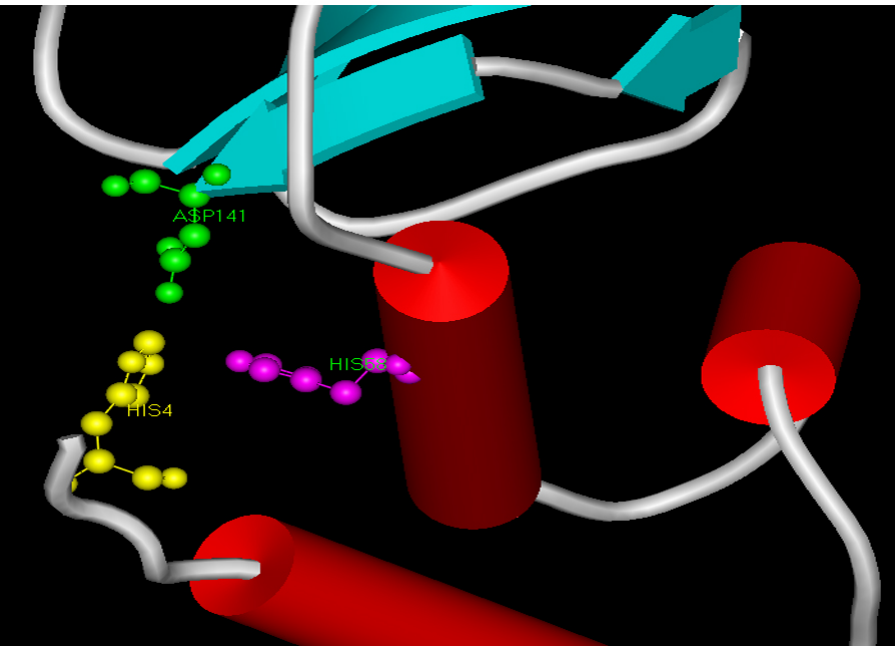
**Metal binding sites of predicted MnSOD of *L. valderian*a BDU20041**. Catalytically essential aspartate or histidine residues are represented in ball and stick mode. Structures are visualized using WebLab ViewerLite 4.2 software [36].

All living systems have only one of each type of SOD in the various cellular compartments indicating that they have far more complex antioxidant defenses than other organisms. As per the findings of Parker and Blake [27], Jackson and Copper [28] and Priya et al [7] our analysis of gene sequence of *L. valderiana *BDU20041 shows that the residues histidine (H_4_, H_58_) and aspartate (D_141_) plays a role in active sites (Figure 8, 9). In addition, the presence of important metal specific residues *viz*., glycine (G_54_), isoleucine (I_81_), glycine (G_106_), tryptophan (W_107_) and proline (P_129_) concludes that the metalloform isolated from *L. valderiana *BDU20041 is that of Mn (Figure 8, 9).

**Figure 10:**
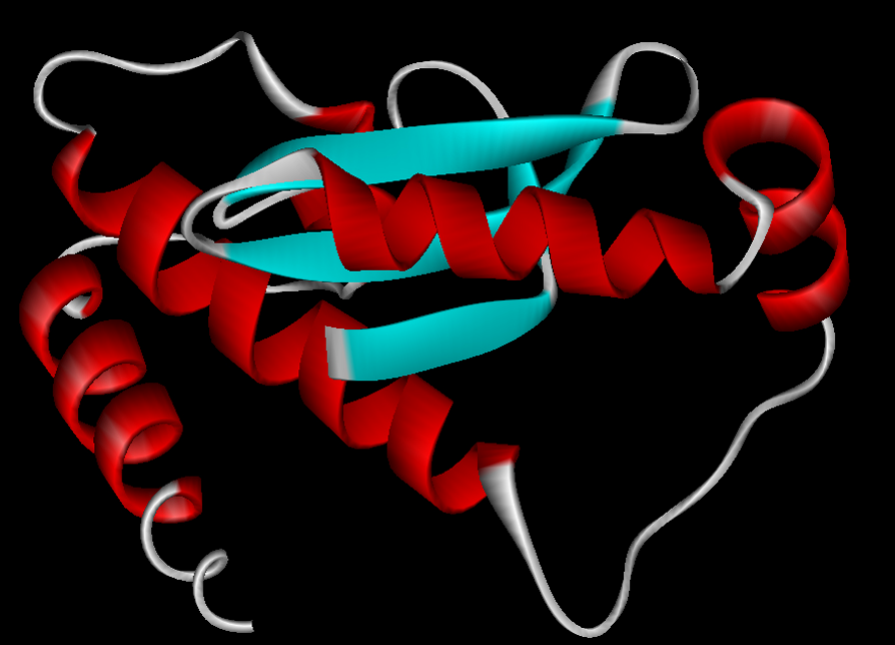
**Predicted structure of MnSOD of *L. valderian*a BDU20041**. Monomeric subunit of MnSOD represents helix (red) and strands (blue). Structures are visualized using WebLab ViewerLite 4.2 software [36].

**Figure 11:**
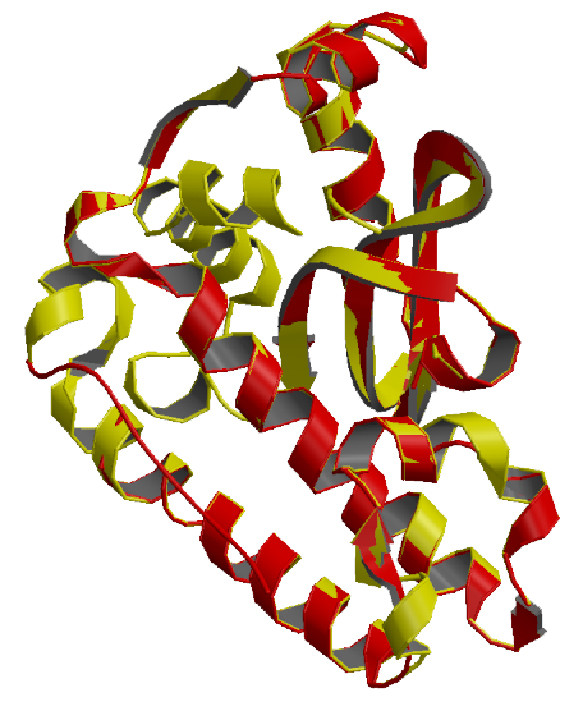
**Superposed structure of MnSOD of *L. valderiana *BDU20041**. Monomeric subunit of MnSOD of *Bacillus anthracis *(1XuQB) depicted red with *L. valderiana *BDU20041 (AAX84682) in yellow sharing 63.8% homology.

The elucidated structure possesses 6 helices and 3 sheets (Figure 10). Homology modeling with MnSOD of *L. valderiana *BDU 20041 showed a similarity of 63.76% with *Bacillus anthracis *(1XUQ) and 60.98% with *Deinococcus radiodurans *(1Y67) (Figure 11). The RMSD value of superposed structure shows that the alpha carbons are at 0.88A and backbone carbon is at 0.85. This further substantiates that the isolated SOD gene is MnSOD with its co-coordinating residues at position His_4_, His_58 _and Asp_141 _(Figure 9).

**Figure 12:**
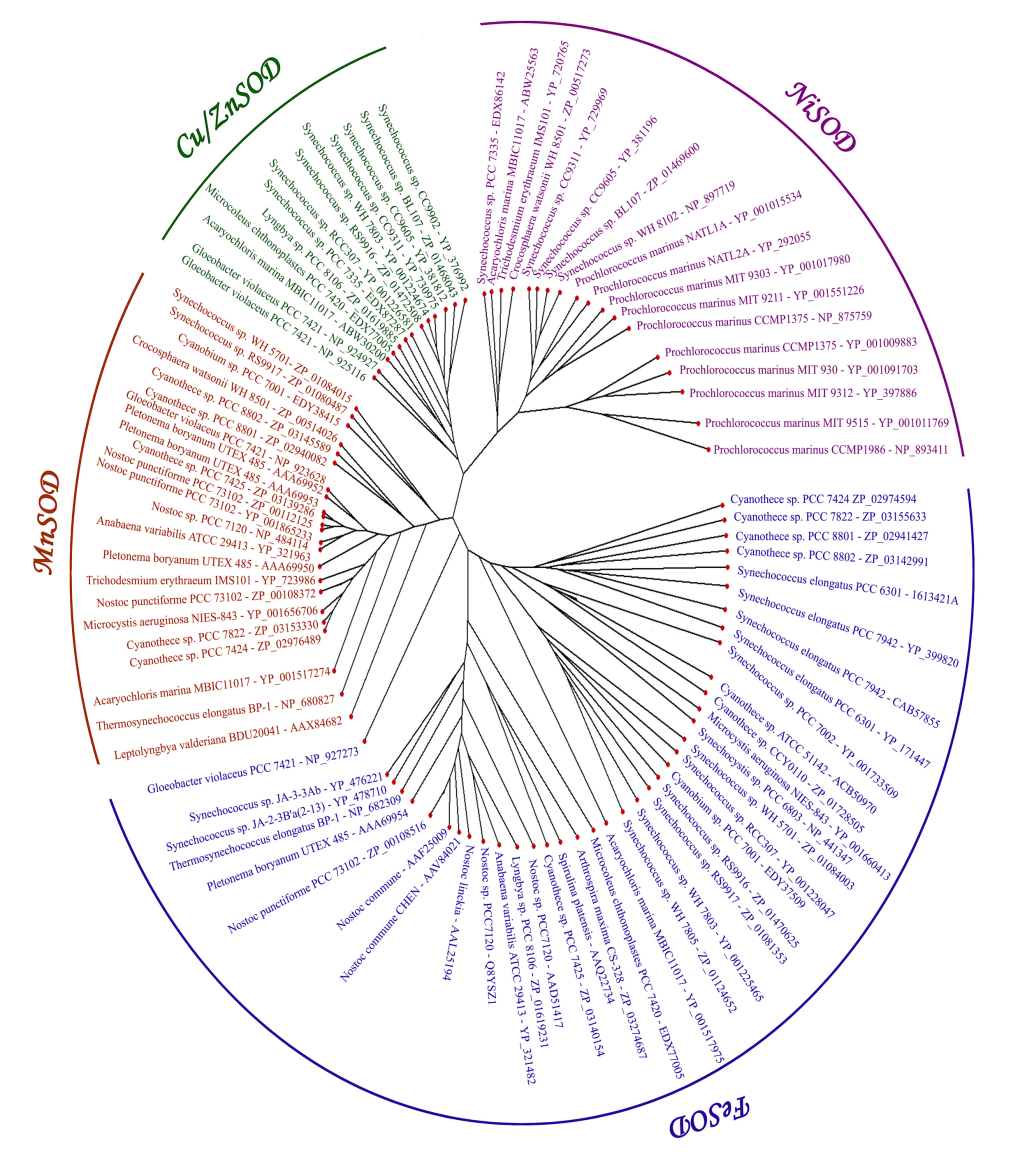
**Phylogenetic tree (radial) of cyanobacterial superoxide dismutases using Neighbour-Joining method using Phylodraw (v.0.8) **[37].

The radial neighbor-joining (NJ) analysis of all cyanobacterial SODs from public database (NCBI/DDBJ/EMBL) showed four distinct metalloforms *viz*., Mn, Fe, Cu/Zn and NiSODs. The SOD gene from *L. valderiana *BDU 20041 is found to be grouped with MnSODs (Figure 12).

The identified MnSOD indicates that it is one of the most probable means through which marine cyanobacterium, *L. valderiana *BDU20041 alleviates oxidative stress caused by abiotic factors (C.I. Acid Black 1).

## Materials and methods

### Azo Dye

The diazo dye, Acid Black 1 (C.I. 20470, Aldrich) was used in the study. Stock solution was prepared by dissolving dye in deionized water and sterilized by membrane filtration (0.2 μM) Millipore.

### Cyanobacterial strain

Axenic culture of *Leptolyngbya valderiana *BDU20041 from the germplasm of National Facility for Marine Cyanobacteria (NFMC), Tiruchrappalli, Tamil Nadu, was utilized in this study. The cells were grown photoautotrophically under continuous illumination with white fluorescent light at about 200 μmol photon m^-2 ^s^-1 ^in 250 mL Erlenmeyer flasks containing artificial sea water (ASN III) medium [29] at 25 ± 2°C for a week.

### Experimental conditions

The medium ASN III [29] was modified as either ASN III nitrogen free medium (without NaNO_3_) or nitrogen limited medium (with a low concentration of 5 mG L^-1 ^NaNO_3_). The organism was grown in ASN III medium for 5 days initially and then in ASN III N limited medium for 5 days at 27 ± 2°C at 20 μmol photon m^2 ^s^-1 ^light intensity, unless otherwise stated. Mid-log cultures were centrifuged; the harvested cells were washed with nitrogen free medium and resuspended in the same medium. This was used as inoculum for all the experiments; the results are presented as means of triplicates.

### Decolourization assay

The decolourization studies were performed by inoculating an equal volume of cultures (500 μL, based on chlorophyll content) to test tubes with 4.5 mL nitrogen free ASNIII medium containing filter sterilized dye, C.I. Acid Black 1 to a final concentration of 0.01%. The cultures were incubated for a stipulated period of 12 days in the above said conditions. Respective abiotic (medium with 0.01% dye only) and biotic (medium with organism only) controls were also maintained in similar conditions as mentioned above.

Culture was harvested by centrifugation at 5,000 g for 10 min on day 0, 1, 2, 6 and 12. The absorption spectrum of the clear supernatant from 400 to 700 *nm *was recorded using a spectrophotometer (JASCO V-550 UV-Visible spectrophotometer, Japan). The percentage of decolourization was calculated as follows

where, A = initial absorbance at zero hour, B = final absorbance.

### Preparation of enzyme extract

Cyanobacterial culture was pelleted by centrifugation at 10,000 × g for 10 min and was washed with ice-cold 25 mM Tris-Cl, pH7.0 and homogenized with the same buffer using Braun sonicator (Labsonic 2000 UB), Germany on an ice bath. Crude lysate was clarified several times by centrifugation at 15,000 *g *for 15 min at 4°C. The clear supernatant obtained served as crude intracellular enzyme source.

### SOD assay

SOD activity was assayed by measuring its ability to inhibit the photochemical reduction of nitroblue tetrazolium (NBT) using the method of Beauchamp and Fridovich [30].

### In vivo detection of ROS using DCFH-DA

The production of Reactive Oxygen Species (ROS) that includes both active oxygen and nitrogen species was detected by using 2',7'-Dichlorofluorescein diacetate (DCFH-DA) in dye (C.I Acid Black 1, 100 mG L^-1^) treated cultures at 3, 24 and 48 hr following He and Hader [14]. DCFH-DA cannot be added or incubated prior to dye treatment due to rapid autooxidation of DCFH-DA or hydrolysed 2, 7-dichlorodihydrofluorescein (DCFH) by exposure to light [15]. Therefore, DCFH-DA (final concentration 5 μM from stock solution of 2 mM) was immediately added to the dye treated and incubated on a shaker at room temperature in dark at 27 ± 2°C for 1 h. The fluorescence of the samples was measured with a spectroflurometer (JASCO V550 spectroflurometer, Japan) at room temperature, with excitation wavelength of 485 *nm *and emission wavelengths at 520 *nm *to determine the relative ROS production. The results were corrected by subtracting the fluorescence of dye untreated control samples.

### DNA isolation

For genomic DNA isolation, actively growing cells were harvested by centrifugation (10,000 × g, 5 min), washed twice with sterile double distilled water followed by a final wash with TE buffer (10 mM Tris: 1 mM EDTA, pH 8.0). Total genomic DNA was extracted using the xanthogenate nucleic acid isolation protocol described by Tillet and Neilan [31].

### PCR amplification and cloning of amplified gene

Oligonucleotide primers corresponding to two separate conserved regions were designed based on the published nucleotide sequence of cyanobacterial Fe/MnSOD and used for PCR amplification of DNA fragment coding for partial SOD gene. The sequence of the primers were 5'-CAC C(C/A)T TGC C(C/T)T A(C/T)G-3' and 5'-GAG GTA GTA AGC (G/A)TG TTC CCA-3'. The PCR reaction was performed using Mastermix (Eppendorf, Germany) containing 1 U *Taq *DNA polymerase, 0.10 μM of each primer, and 50 nG of template DNA in a 25.0 μL reaction mixture using a Master gradient thermal cycler (Eppendorf, Germany). The cycling profile included an initial denaturation at 94°C for 3 minutes followed by 29 cycles of 94°C for 1 min, 53°C for 1 min (gradient, G = 10°C), 72°C for 1 min, and a final extension at 72°C for 6 minutes. Sterile water replaced DNA for the control. Five microliters of the amplified products were subjected to 1.5% agarose gel electrophoresis containing ethidium bromide (2.25 mg ml^-1 ^distilled water) in 1× TAE (40 mM Tris-acetate: 1 mM EDTA), visualized with a UV transilluminator and documented using CCD camera (Alpha Imager™ 2200, USA).

The resulting amplicons was purified with the Qiagen gel extraction kit (Qiagen GmbH, Hilden, Germany) and subcloned into the pGEM-T vector (Promega, Madison, WI) following the directions of the manufacturer. The construct was transformed into DH5α competent cells using electroporation with 1800V, 25 microfarads, and 200 *ohms *in a Gene Pulser II electroporation system (Bio-Rad).

### DNA sequencing

Clones were sequenced using M13 forward and reverse oligonucleotide primers (Integrated DNA Technologies, USA) for the DNA cycle sequencing reaction kit following recommended protocol. The DNA sequences of the amplified product were obtained with an ABI 310 automated sequencer using the chain-termination method with big-dye terminators (Applied Biosystems, Foster, CA, USA). For the sequence data, automated base calls were checked by manual inspection of the electropherograms of both forward and reverse sequences. The base call conflicts were resolved by aligning and comparing both strands using SeqScape^® ^software v 2.5 (Applied Biosystems, Foster, CA, USA).

### Characterization of isolated SOD gene

The computational analysis of the isolated gene was carried out using protein analysis [32], sequence alignment (Clustal W) of BioEdit [33], domain pattern [34], homology modeling and analysis [35] and visualized using WebLab ViewerLite [36]. Phylogenetic tree was constructed using multiple alignments and visualized in Phylodraw v.0.8 [37].

### Nucleotide Sequence Accession Numbers

The nucleotide sequence of the SOD gene has been deposited in NCBI under accession no. AY974247.

## Competing interests

The authors declare that they have no competing interests.

## Authors' contributions

BP and KRS carried out the molecular genetic studies, participated in the sequence alignment and drafted the manuscript. VDJ helped in carrying out the structural comparison. GS helped in fine tuning of the manuscript. LU and DP conceived of the study and participated in its design and coordination. All authors read and approved the final manuscript.

## References

[B1] Atzenhofer W Regelsberger G Jacob U Peschek G Furtmuller P Huber R Obinger C The 2.0A resolution structure of the catalytic portion of a cyanobacterial membrane-bound manganese superoxide dismutase J Mol Biol 2002 321 479 489 10.1016/S0022-2836(02)00624-1 12162960

[B2] van Baalen C Quantitative surface plating of coccoid blue-green algae J Phycol 1965 1 19 22 10.1111/j.1529-8817.1965.tb04550.x

[B3] Patterson C Myers J Photosynthetic production of hydrogen peroxide by *Anacystis nidulans* Plant Physiol 1973 51 104 109 10.1104/pp.51.1.104 16658269 PMC367365

[B4] Morales I Bateucas S de la Rosa FF Storage of solar energy by production of hydrogen peroxide by the blue-green alga *Anacystis nidulans *R2: stimulation by azide Biotechnol Bioeng 1992 40 147 150 10.1002/bit.260400120 18601055

[B5] Kalavathi DF Uma L Subramanian G Degradation and metabolization of the pigment-melanoidin in distillery effluent by the marine cyanobacterium *Oscillatoria boryana *BDU 92181 Enz Microbial Technol 2001 29 246 251 10.1016/S0141-0229(01)00383-0

[B6] Herbert SK Samson G Fork DC Laudenbach DE Characterization of damage to photosystems I and II in a cyanobacterium lacking detectable iron superoxide dismutase activity Proc Natl Acad Sci USA 1992 89 8716 8720 10.1073/pnas.89.18.8716 1528884 PMC49991

[B7] Priya B Premanandh J Dhanalakshmi TR Uma L Prabaharan D Subramanian G Comparative analysis of cyanobacterial superoxide dismutases to discriminate canonical forms BMC Genomics 2007 8 435 444 10.1186/1471-2164-8-435 18042279 PMC2234264

[B8] Thomas DJ Avenson TJ Thomas JB Herbert SK A cyanobacterium lacking iron superoxide dismutase is sensitized to oxidative stress induced with methyl viologen but is not sensitized to oxidative stress induced with norflurazon Plant Physiol 1998 116 1593 602 10.1104/pp.116.4.1593 9536078 PMC35068

[B9] Fridovich I Superoxide dismutases. An adaptation to a paramagnetic gas J Biol Chem 1989 264 7761 4 2542241

[B10] Swaminathan P Prabaharan D Uma L Fate of few pesticide-metabolizing enzymes in the marine cyanobacterium *Phormidium valderianum *BDU 20041 in perspective with chlorpyrifos exposure Pesticide Biochem Physiol 2009 94 68 72 10.1016/j.pestbp.2009.03.003

[B11] Swaminathan P Saha SK Uma L Laccase and Poly phenol oxidase activities of marine cyanobacteria, a study with Poly R-478 decolourization World J Microbiol Biotechnol 2010 26 63 69 10.1007/s11274-009-0143-y

[B12] Saha SK Uma L Subramanian G Nitrogen stress induced changes in the marine cyanobacterium *Oscillatoria willei *BDU 130511 FEMS Microbiol Ecol 2003 45 263 272 10.1016/S0168-6496(03)00162-4 19719595

[B13] Subramanian G Uma L Priya B Prabaharan D Utilization of microalgae to address pollution problems Indian Hydrobiol 2007 10 1 25

[B14] Priya B Marine Cyanobacteria - a plausible candidate for biodecolourization of textile dye: with emphasis on AOS Ph.D Dissertation 2009 Bharathidasan University, Tiruchirappalli, India

[B15] He YY Häder D Reactive oxygen species and UV-B: effect on cyanobacteria Photochem Photobiol Sci 2002 1 729 36 10.1039/b110365m 12656470

[B16] Bhattacharjee S Reactive oxygen species and oxidative burst: Roles in stress, senescence and signal transduction in plants Curr Sci 2005 89 1113 1121

[B17] Parikh A Madamwar D Textile dye decolorization using cyanobacteria Biotechnol Lett 2005 27 323 326 10.1007/s10529-005-0691-7 15834793

[B18] Omer HH Algal decolourization and degradation of monoazo and diazo dyes Pakistan J of Biol Sci 2008 11 1310 131 10.3923/pjbs.2008.1310.1316 18817261

[B19] Asada K Foyer CH, Mullineaux PM Production and action of active oxygen species in photosynthetic tissues Causes of Photooxidative Stress and Amelioration of Defense Systems in Plants 1994 Florida, CRC Press 77 104

[B20] Henzler T Ye Q Steudle E Oxidative gating of water channels (aquaporins) in *Chara *by hydroxyl radicals Plant Cell and Environ 2004 27 1184 1195 10.1111/j.1365-3040.2004.01226.x

[B21] Hirano T Tanaka H Enoki A Relationship between production of hydroxyl radicals and degradation of wood by the brown-rot fungus, *Tyromyces palustris* Holzforschung 1997 51 389 95 10.1515/hfsg.1997.51.5.389

[B22] Tanaka H Itakura S Enoki A Hydroxyl radical generation by an extracellular low-molecular-weight substance and phenol oxidase activity during wood degradation by the white-rot basidiomycete *Trametes versicolor* J Biotechnol 1999 75 57 70 10.1016/S0168-1656(99)00138-8 10704993

[B23] Joseph JM Destaillats H Hung HM Hoffmann MR The sonochemical degradation of azobenzene and related azo dyes: Rate Enhancements *via *Fenton's Reactions J Phys Chem 2000 104 301 307

[B24] Latifi A Ruiz M Zhang C Oxidative stress in cyanobacteria FEMS Microbiol Rev 2009 33 258 278 10.1111/j.1574-6976.2008.00134.x 18834454

[B25] Karpinski S Reynold H Karpinska B Wingsle G Creissen G Mullineaux P Systemic signalling and acclimation in response to excess excitation energy in *Arabidopsis* Science 1999 284 654 657 10.1126/science.284.5414.654 10213690

[B26] Polle A Dissecting the superoxide dismutase-ascorbate-glutathione-pathway in chloroplasts by metabolicmodeling Plant Physiol 2001 126 445 462 10.1104/pp.126.1.445 11351106 PMC102317

[B27] Parker WM Blake CFC Iron and manganese containing superoxide dismutases can be distinguished by analysis of their primary structures Federation of European Biochemical societies 1988 229 377 382 10.1016/0014-5793(88)81160-8 3345848

[B28] Jackson SMJ Cooper JB An analysis of structural similarity in the iron and manganese superoxide dismutases based on known structures and sequences Biometals 1998 11 159 173 10.1023/A:1009238214394 9542069

[B29] Rippka R Deruelles J Waterbury JB Herdman M Stanier RY Generic assignments, strain histories and properties of pure cultures of cyanobacteria J General Microbiol 1979 111 1 61

[B30] Beauchamp C Fridovich I Superoxide dismutase: improved assays and an assay applicable to acrylamide gels Anal Biochem 1997 144 276 287 10.1016/0003-2697(71)90370-8 4943714

[B31] Tillett D Neilan BA Xanthogenate nucleic acid isolation from cultured and environmental cyanobacteria J Phycol 2000 36 251 258 10.1046/j.1529-8817.2000.99079.x

[B32] Gasteiger E Gattiker A Hoogland C Ivanyi I Appel RD Bairoch A ExPASy: the proteomics server for in-depth protein knowledge and analysis Nucleic Acids Res 2003 31 3784 3788 10.1093/nar/gkg563 12824418 PMC168970

[B33] Hall TA BioEdit: a user-friendly biological sequence alignment editor and analysis program for Windows 95/98/NT Nucl Acids Symp Ser 2005 41 95 98

[B34] Pagni M Ioannidis V Cerutti L Zahn-Zabal M Jongeneel CV Falquet L MyHits: a new interactive resource for protein annotation and domain identification Nucleic Acids Res 2004 1 32 10.1093/nar/gkh479 PMC441617 15215405

[B35] Guex N Peitsch MC SWISS-MODEL and the Swiss-PdbViewer: An environment for comparative protein modeling Electrophoresis 1997 18 2714 2723 10.1002/elps.1150181505 9504803

[B36] WebLab ViewerLite software http://in.msi.com/

[B37] Phylodraw http://pearl.cs.pusan.ac.kr/phylodraw/

